# The Psychrotolerant Antarctic Fungus *Lecanicillium muscarium* CCFEE 5003: A Powerful Producer of Cold-Tolerant Chitinolytic Enzymes

**DOI:** 10.3390/molecules21040447

**Published:** 2016-04-05

**Authors:** Massimiliano Fenice

**Affiliations:** Dipartimento di Scienze Ecologiche e Biologiche, University of Tuscia, Largo Università snc, I-01100 Viterbo, Italy; fenice@unitus.it; Tel.: +39-0761-357-318

**Keywords:** Continental Antarctica, chitinolytic enzymes, *Lecanicillium muscarium*, cold-tolerant enzymes

## Abstract

*Lecanicillium muscarium* CCFEE 5003, isolated in Continental Antarctica, is a powerful producer of extracellular cold-tolerant enzymes. Chitin-hydrolyzing enzymes seems to be the principal extracellular catalytic activities of this psychrotolerant fungus. The production of chitinolytic activities is induced by chitin and other polysaccharides and is submitted to catabolite repression. The chitinolytic system of *L. muscarium* consists of a number of different proteins having various molecular weights and diverse biochemical characteristics, but their most significant trait is the marked cold-tolerance. *L. muscarium* and selected strains of the biocontrol agent of pathogenic fungi *Trichoderma harzianum*, have been compared for their ability to produce chitinolytic enzymes at different temperatures. At low temperatures the Antarctic strain was definitely much more efficient. Moreover, the fungus was able to exert a strong mycoparasitic action against various other fungi and oomycetes at low temperatures. The parasitic role of this organism appeared related to the production of cell wall degrading enzymes being the release of extracellular chitinolytic enzymes a key event in the mycoparasitic process. Due to the mentioned characteristics, *L. muscarium* could have an important role for potential applications such as the degradation of chitin-rich materials at low temperature and the biocontrol of pathogenic organisms in cold environments. For these reasons and in view of future industrial application, the production of chitinolytic enzymes by the Antarctic fungus has been up-scaled and optimised in bench-top bioreactor.

## 1. Introduction

Antarctica is generally considered the most extreme continent concerning temperature, winds, altitude, isolation, UV-irradiation and dryness [1]. The very unhospitable Antarctic environment requires high levels of adaptation and specialization. Adaptation is the fundamental topic in Antarctic biology and the study of the biodiversity of Antarctic organisms is of paramount scientific importance: Antarctica is a unique and precious source of genes that are only partially investigated [2].

Microorganisms dominate the Antarctic biology [3]. What adaptation strategies do Antarctic microorganisms adopt? They must cope with combinations of highly stressful environmental conditions and in particular low temperatures, wide temperature fluctuations, scarce water availability, frequent freeze-thaw cycles and, in summer, intense solar radiation [4]. Most of them show physiological parameters optima well above the average environmental temperature [4,5], even if they still function close to freezing conditions where they are subjected to great increase of external solutes concentration needing resistance to osmotic stress for survival [6]. Other adaptation strategies include pigmentation to face high levels of radiation and peculiar fatty acid compositions to increase membrane fluidity [7,8]. 

The majority of Antarctic microfungi are psychrotolerant instead of psychrophilic [5,9], showing better adaptation to the unstable environment. Here, microorganisms must grow whenever water is available and the production of enzymes having broad temperature range of activity would be a winning survival strategy.

Chitinolytic enzymes have been widely studied and some of them, produced by fungi, appear of great interest for industrial or environmental applications. Nevertheless, there is still necessity of cheap and commercially suitable sources of these enzymes.

**Figure 1:**
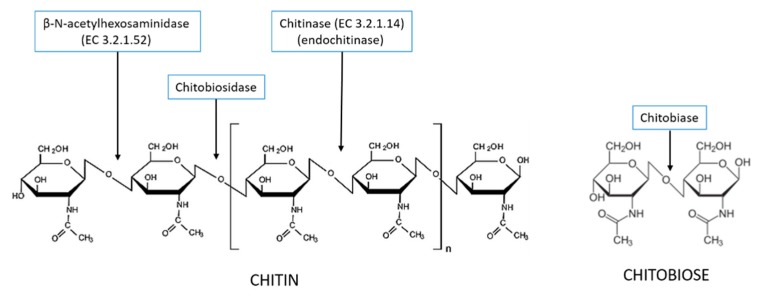
Schematic representation of the catalytic activity by some chitinolytic enzymes on chitin and chitobiose. Only chitinase (EC 3.2.1.14) and β-N-acetylhexosaminidase (EC 3.2.1.52) are described by the Enzyme Commission.

The hydrolysis of chitin is performed by a series of chitinolytic enzymes that are generally called chitinases using a misleading term. Their current classification, supplied by the Enzyme Commission (EC), is inadequate to describe the various and diverse activities found in Nature, having very different hydrolytic action on the polysaccharide [10]. Only two classes of enzymes are listed, being the different activities now merged in the same EC class: chitinase, (EC 3.2.1.14) producing “random hydrolysis of *N*-acetyl-β-d-glucosaminide (1 → 4)-β-linkages in chitin and chitodextrins”; and β-*N*-acetylhexosaminidase (EC 3.2.1.52) that “releases N-acetyl-d-hexosamine residues, at the non-reducing terminal, from chitin and chitodextrins”. However, numerous authors still use the old classification making the actual situation rather confusing and ambiguous [11,12]. The enzymes acting randomly inside the polysaccharide, generating shorter fragments, are frequently defined as “endo-chitinases”, while those acting externally are defined as “exo-chitinases” [13,14,15]. Moreover, other much specific hydrolytic mechanisms had been described (Figure 1). For example, enzymes acting only on chitobiose are called “chitobiases”, while those releasing this disaccharide from chitin or chitodextrins are named “chitobiosidases” [13,16].

Conventionally, most of these hydrolases find uses in chitin hydrolysis (including the treatment of chitin-rich wastes), production of chitin derivatives, protoplast formation and biocontrol of pathogenic organisms [17,18,19,20,21]. Moreover, some innovative applications in the food and wine industries have been successfully tested at the laboratory level [21,22]. Nevertheless, most of the abovementioned processes, in particular those concerning environmental problems (*i.e.*, on-field control of pathogens or degradation of chitin-rich wastes), are strongly restricted by adverse environmental conditions, in particular low temperature, affecting both the chitinolytic organisms and their hydrolytic enzymes. This is a notorious problem for recognised powerful chitinolytic organisms such as *Trichoderma harzianum* [19,23] even if, recently, some strains were shown to be active at 10 °C [24]*.* Thus, the availability of a psychrophilic or, better, a psychrotolerant organism, able to release cold-active chitinolytic enzymes at very low temperature (5 °C or below), is important where other microorganisms fail. This is particular true in many applications including the hydrolysis of chitin-rich wastes at low temperature and the biocontrol of pathogens in cold environments. In this context, the search of new chitinolytic organisms and/or enzymes is still of great scientific and applied interest [25].

Among chitinolytic fungi, the genus *Lecanicillium* has been mainly studied for its involvement in the pathogenesis of a wide array of invertebrates; “Lecanicilli” have a wide host range and have been isolated from a variety of insects [26]. *Lecanicillium muscarium*, a good representative of this genus, has also been isolated from insects (*i.e.*, aphids, scales, whiteflies, thrips, planthoppers, *etc.*) all over the world and its pathogenic activity against them proved [27,28,29,30,31,32,33,34]. Preparations containing the fungus, active against whiteflies, thrips, aphids and mites, are commercialised worldwide as the bio-pesticides “Mycotal” and “Verticillin” [35]. *L. muscarium* has also been isolated from arachnids and nematodes [31,36]. The fungus exerts also its parasitic activity against various fungi involved in plant diseases and its use as biocontrol agent has been demonstrated [31,37,38,39,40]. The mycoparasitism against oomycetes has been demonstrated “*in vitro*” as well [19].

In this study, we will review the fascinating features of an emerging strain (CCFEE 5003) of *Lecanicillium muscarium* isolated in Continental Antarctica and showing very high production of a wide array of extracellular cold-active chitinolytic enzymes. 

## 2. *Lecanicillium muscarium* CCFEE 5003

*Lecanicillium muscarium* (Petch) strain CCFEE 5003 Zare & W. Gams was previously affiliated to *Acremonium strictum* W. Gams [5] then to *Verticillium lecanii* Zimm strain A3, [17]. The new genus *Lecanicillium* W. Gams & Zare, was proposed to accommodate the majority of entomogenous or fungicolous *Verticillium* (section *Prostrata*) species [41]. A detailed account of the species and the new combinations are supplied by Zare and Gams [26].

*Lecanicillium muscarium* CCFEE 5003, isolated from moss collected in Victoria Land on the west coast of the Ross Sea (Antarctica) [5], is stored in the Culture Collection of Fungi from Extreme Environments (CCFEE:) of the Dipartimento di Scienze Ecologiche e Biologiche, University of Tuscia, Viterbo, Italy. 

The thermal preferences of the fungus showed its clear psychrotolerant behaviour, being its optimum of growth at 25 °C with no big differences within 20–28 °C. The fungus was also able to grow, albeit much slower, at 0 °C. It is worth noting that *L. muscarium* sporulation occurred in the whole temperature range from 0 to 28 °C, showing its great ability to diffuse in the environment [5].

During a preliminary study, screening the production of extracellular enzymes by Antarctic fungi, strain CCFEE 5003 was noted for its production of a number of hydrolases (amylases, cellulases, proteases and lipases) but was selected for its high chitinolytic activity (*ca.* 300 IU/L, International Units) in submerged shaken cultures [17,42].

## 3. Production and Characterization of Chitinolytic Enzymes by *L. muscarium* CCFEE 5003

The production of chitinolytic enzymes by *L. muscarium*, in shaken cultures, appeared to be inducible by various forms of raw and purified chitin [17,19,43]. Even if to a lesser extent, induction was also obtained with glucans but, on the other hand, glucanases were also produced when the fungus grew on chitins, being the production of these two cell wall degrading enzymes (CWDE) always correlated [19]. The production of chitinolytic enzymes appeared to be subjected to catabolite repression: no activity was detected in media containing both glucose and chitin until complete glucose depletion occurred. In addition, no activity was found in media containing glucose as the sole carbon source. The chitinolytic enzymes of *L. muscarium* were mainly present inside the fungal cells during the first 24 h of growth to be massively secreted thereafter [17]. Strain CCFEE 5003 was also able to grow and release high levels of these enzymes in media containing crab or shrimp wastes. This characteristic is particularly interesting in view of applications at the environmental/industrial level [10].

**Figure 2:**
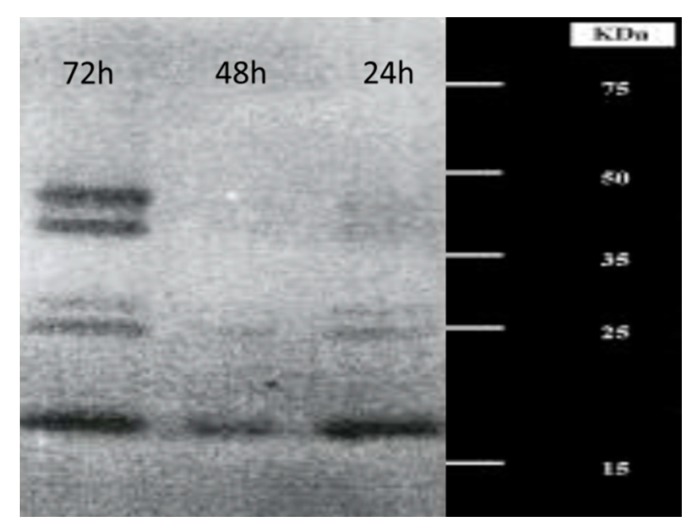
One-dimension activity PAGE for chitinolytic enzymes by *Lecanicillium muscarium* CCFEE 5003 produced in shaken cultures at different times: 24 h, intracellular activity; 48 and 72 h extracellular activity. Molecular weight markers are shown in the range 15–75 kDa (unpublished picture related to [19]).

The enzymatic system of *L. muscarium*, related to chitin hydrolysis, is very complex and has been studied in detail. In shaken flasks, the fungus was able to release at least five different enzymes as revealed by electrophoresis polyacrylamide gel (PAGE) carried out under partially denaturing conditions, followed by renaturation and stained for activity using glycol chitin as substrate. These proteins showed molecular weights ranging from 20 to 45 kDa (Figure 2) and isoelectric points in the range 4.5–5.5 pH [19]. Moreover, a chitobiose-hydrolysing protein, not revealed by the activity-gel, was released [10]. The principal chitinolytic enzymes produced by *L. muscarium* were purified and biochemically characterised; all of them were also very active at low temperature [10,43]. A complex enzymatic system, consisting of many different proteins, having complementary action on chitin, is typical of very aggressive fungi using their enzymes to attack other organisms. Others, using environmental chitin merely as a nutrient, could have a much simplified system with just one or few proteins. 

The first enzyme studied in the early works (CHI) [43] was purified by preparative isoelectric focusing and ion-exchange chromatography on Q-Sepharose. It was able to hydrolyse colloidal and glycol chitin better than other forms of chitin; xylan and chitosan were scarcely degraded. Moreover, no activity was detected on cellulose and chitobiose. This protein (molecular weight of 45 kDa, in SDS-PAGE) appeared to be glycosylated and had an isoelectric point of 4.9. The optimal pH for its activity was 4.0; at pH 3.5 and 4.5 the enzyme retained 53% and 66% of the activity shown at the optimum, respectively. Enzyme stability was highest at pH 5.0. The purified enzyme was active from 5 to 60 °C with maximum at 40 °C. It is worth noting that at 5 °C, 50% of its maximum activity was still retained. In addition, strong inhibition by Zn^2+^, Mn^2+^, Mg^2+^ and EDTA (100 mM) was recorded.

Two other chitinolytic enzymes, CHI1 and CHI2, were purified and biochemically characterized [10]. CHI1, having molecular weight of 61 kDa, showed optima at pH 5.5 and 45 °C; while CHI2, having molecular weight of 25 kDa, showed optima at pH 4.5 and 40 °C. Both enzymes maintained high levels of activity at 5 °C and were inhibited by Fe^2+^, Hg^2+^ and Cu^2+^. In addition, CHI2 was markedly sensitive to allosamidin. Both proteins could be classified as *N*-acetylhexosaminidases (E.C. 3.2.1.52), but showed different roles in chitin hydrolysis: CHI1 could be defined as a “chitobiase” while CHI2 revealed a main “exo-chitinase” activity [10].

The preliminary molecular characterization of a gene coding for a chitinase (probably CHI) from *L. muscarium* CCFEE 5003 has been obtained sequencing a 736 bp DNA fragment [44]. This corresponded to 245 amino acids and represented *ca.* 60% of the whole protein sequence for the *Lecanicillium* spp. endochitinase (GH18, glycosyl hydrolases, family 18, 423 aa). However, according to the comparison carried out in the NCBI GenBank database with BlastX, no match with *Lecanicillium* chitinolytic enzymes was obtained. By contrast, matching was achieved with various other fungal chitinases (Table 1). It is worth noting that, in all cases, although the query coverage was quite high (93%–95%), the identities were always low (never exceeding 50%). These marked differences could be explained as an adaptation strategy adopted by the Antarctic fungus to produce enzymes active in a broad temperature range.

## 4. Potential Applications of the Chitinolytic Enzymes by *L. muscarium* CCFEE 5003

Since cell walls of most pathogenic or spoiling microorganisms contain chitin, the degradation of which affects their growth and differentiation, chitinolytic enzymes could play a fundamental role in the control of these organisms [43]. The above mentioned features suggested possible applications of the Antarctic strain and its enzymes.

**Figure 3:**
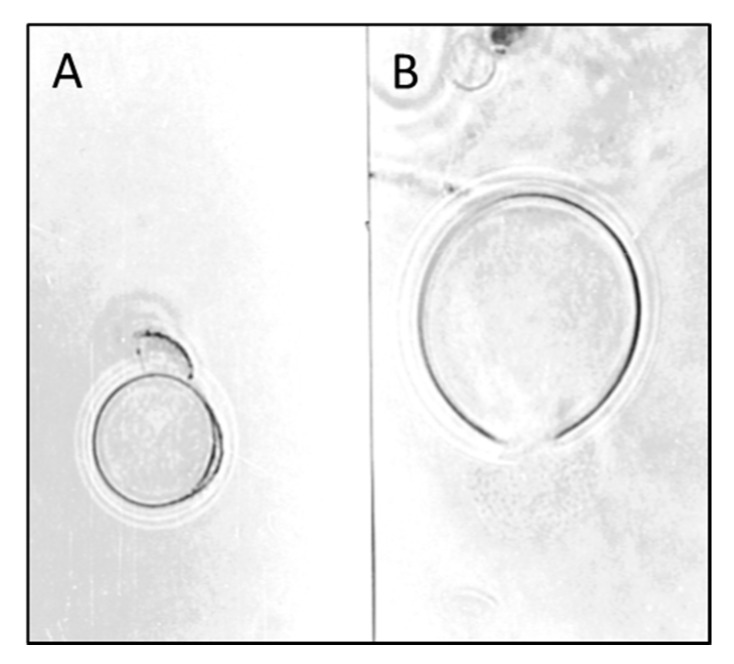
Cultures of *Mucor plumbeus* treated with the purified chitinolytic enzyme (CHI) of *L. muscarium* CCFEE 5003 and showing protoplast formation, with traces of degraded cell wall, (**A**) and bursting (**B**) (unpublished picture related to [43]).

The purified CHI showed evident inhibitory effects on a series of moulds involved in refrigerated food spoilage (*Mucor plumbeus*, *Cladosporium cladosporoides*, *Penicillium verrucosum* and *Aspergillus versicolor*)*.* The enzyme caused mycelial damages, cell lysis, inhibition of conidia germination, formation and bursting of protoplast (Figure 3). The results of this study would suggest that the chitinolytic enzyme could be employed in place or in combination with traditional food preservatives, the use of which has been widely criticized [43], as already proposed in food technology for other enzymatic preparations containing chitinolytic enzymes [22].

The use of purified enzymes in large scale applications is expensive and sometime not necessary, while crude or semi-purified enzyme preparations could represent a feasible solution. The crude CWDE produced by *L. muscarium* have been applied on red and white grape bunches inoculated with spores of *Aspergillus carbonarius*, a well-known producer of ochratoxin A, in order to verify possible reduction of the contamination by the toxigenic fungus in post-harvest conditions. Presence of the contaminant in the control (untreated grapes) was very high (>2000 CFU/mL); by contrast, on treated fruits, it was reduced by 89 and 95%, for red and white grape, respectively. Light microscopy showed that the enzymes solution induced in *A. carbonarius* spores the same damages described in Figure 3 [21].

**Figure 4:**
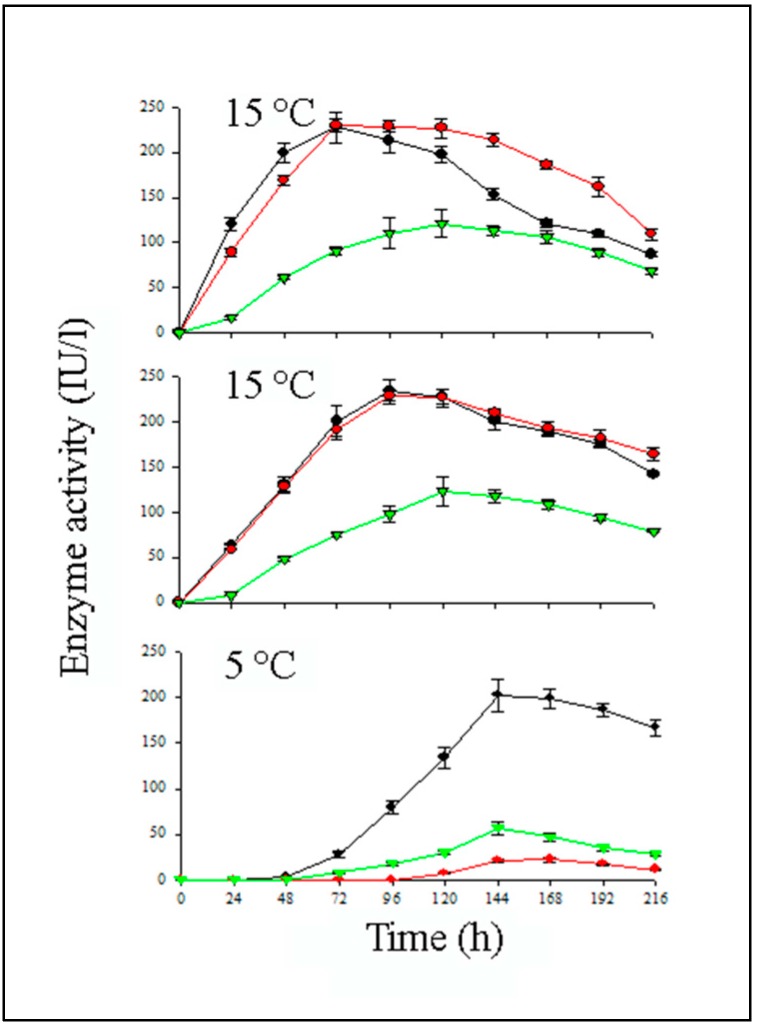
Chitinolytic activity of *L. muscarium* CCFEE 5003 (**black lines**), *T. harzianum* P1 (**green lines**) and *T. harzianum* T22 (**red lines**) at 5, 15 and 25 °C. (Modified from [43]).

The high cold tolerance of *L. muscarium* and its enzymes could be exploited in view of possible applications as a biocontrol organism for phytopathogens in cold environments. Thus, the fungus was compared with two selected strains of *Trichoderma harzianum* (strains P1 and T22), a well-known chitinolytic fungus commercially used for the above applications but showing limitations at low temperatures [23,45]. The three strains were grown at 5, 15 and 25 °C under inducing conditions (Figure 4).

At 25 °C, for both *L. muscarium* and *T. harzianum* T22, the time course of the enzyme activity was quite similar, reaching a maximum (*ca* 230 IU/L) after 72 h of incubation. Strain P1 performance was definitely lower reaching only 121 IU/L after 120 h. At 15 °C, the performance of the three strains was quite similar to that recorded at 25 °C. At 5 °C, the enzyme activity of *L. muscarium* was very similar to that obtained at 25 °C (203 IU/L after 144 h). On the contrary, the production of the two *Trichoderma* strains was definitely lower (22 and 57 IU/L, for strain T22 and P1, respectively) [43].

## 5. Optimization of the Chitinolytic Enzymes Production in Bioreactors

In view of possible applications at the industrial level, microorganisms must be cultivated in bulk. Thus, every possible industrial strain must show good capability to grow and produce the studied enzymes and/or metabolites in bioreactors. Not all microorganisms possess this characteristic that is mandatory for every process scale-up.

In this context, the production of chitinolytic enzymes by strain CCFEE 5003, after preliminary tests in shaken flasks, was optimised in bench-top bioreactors using both purified chitin (colloidal) and chitin-rich materials obtained from crab and shrimp shells wastes.

**Figure 5:**
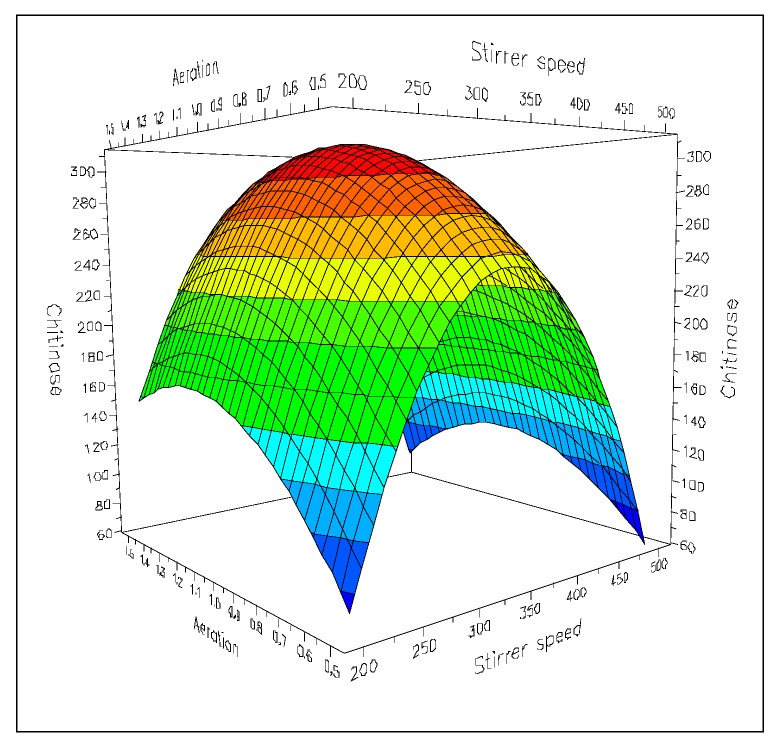
Combined effects of agitation and aeration on the chitinolytic activity of *L. muscarium* CCFEE 5003 cultivated in bioreactor as predicted by RSM. Colour codes in the surface response indicate different ranges of values (unpublished picture related to [46]).

Response Surface Methodology (RSM) was employed to investigate the effects of combining stirrer speed (in the range 200–500 rpm) and aeration rate (in the range 0.5–1.5 vvm) [46]. Optimization of these fundamental process parameters was carried out using quantitative and quantitative-multilevel factors for aeration and agitation, respectively. The model (quadratic D-optimal) revealed high reliability and good statistical performance (*R*^2^, 0.931; *Q*^2^, 0.869). Maximum of activity (373.0 IU/L), under optimised conditions, was predicted at an intermediate stirring rate (*ca*. 327 rpm) and 1.1 vvm. However, the subsequent experimental confirmation of the model indicated that highest activity (383.7 IU/L) was achieved at 1 vvm and 300 rpm. Clearly, the enzyme production was strongly affected by the shear effects produced by high agitation and aeration rates (Figure 5). In bioreactors, under optimised conditions, the fungus produced a number of chitinolytic enzymes higher than those released in shaken flasks; moreover, production was 23% higher.

This work, demonstrating the capacity of *L. muscarium* CCFEE 5003 to grow in bench-top bioreactors, identified the optimal conditions for the possible upscale of production at the industrial level [46].

On raw wastes from shrimp and crab manufacture, the production of chitinolytic enzymes by the fungus (in shaken flasks) was much lower than that obtained using colloidal chitin. This was an expected result, since colloidal chitin is a purified substrate with stronger inducing effects. In addition, the two wastes contained traces of other nutrients, such as fats and proteins, that could activate some repression mechanisms. However, since the production on shrimp wastes was much higher than that on crab shells (104.6 and 48.6 IU/L, respectively) optimization in bioreactor was carried out using the first substrate only. In this case, optimization in bioreactor by RSM was carried out to find best pH and substrate concentration under the optimised conditions of agitation and aeration previously reported. The optimization process permitted to improve the production by 137% (243.6 IU/L).

The ability of strain CCFEE 5003 to produce high level of chitinolytic enzymes in submerged processes was not accompanied by same result when the fungus was grown in solid-state bioreactors (unpublished results). This was somehow an unexpected result since Lecanicilli are known to be quite good producers of these enzymes also when employing this technology [18,47].

## 6. Mycoparasitic Action of *L. muscarium*

Bruce and co-workers [48] described mycoparasitism as an antagonistic interaction between two fungal organisms during which the parasite establishes intimate contacts with the host prior to release CWDE. Other authors [49,50,51] correlated mycoparasitism to well-defined events, such as coiling around and penetration into the host, also mentioning the release of lytic enzymes. Nevertheless, most authors agree that stable contacts between the two organisms must happen [49,50,51]. Some mycoparasites (*i.e.*, *Trichoderma* spp.) produce extracellular inhibiting metabolites and/or volatile compounds with no physical contact with the host [51].

*L. muscarium* CCFEE 5003 always established firm contact with the host mycelia after an initial phase during which inhibitory compounds were probably released. In all cases, the contact with the host caused its disruption.

The mycoparasitism of the Antarctic strain occurred in a wide range of temperatures (5–25 °C) and it was tested “*in vitro*” through plate co-cultures with a series of moulds (*Aspergillus versicolor*, *Botrytis cinerea*, *Cladosporium cladosporoides*, *Penicillium verrucosum*, *Mucor mucedo* and *M. plumbeus*) and oomycetes (*Pythium aphanidermatum* and *Phytophthora palmivora*) causing food spoilage or phytopathogenicity [19,43].

**Figure 6:**
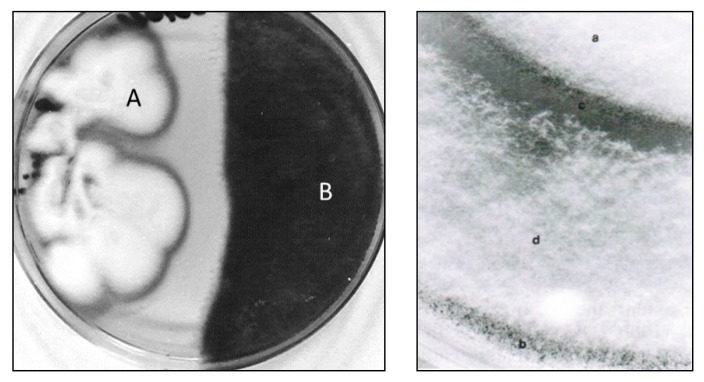
Left: dual culture of *L. muscarium* (**A**) and *Botrytis cinerea* (**B**). Right: detail of a dual culture of *L. muscarium* (**a**) and *Mucor plumbeus* (**b**); (**c**) contact zone between the two fungi; (**d**) overgrowth of *L. muscarium* on *M. plumbeus* (Right: unpublished picture related to [43]. Left: picture modified from [43]).

**Figure 7:**
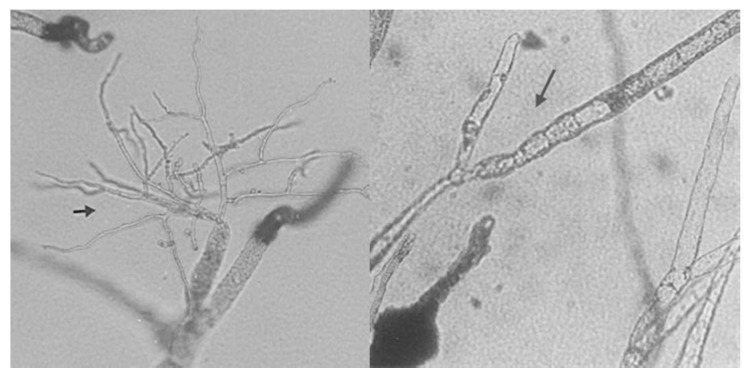
Branching (**left**) and vacuolated (**right**) mycelia of *Mucor mucedo*. Grown in dual culture with *L. muscarium* (modified from [19]).

In all co-cultures, common features were recorded. The possible hosts grew well up to 3–10 mm from *L. muscarium* colonies, when entered an “inhibition zone” (Figure 6). At this phase, all host organisms terminated their growth while various alterations occurred to their mycelia. Mycelial alterations: (branching, vacuolation, cell-wall damages and presence of abnormal structures) were never observed in axenic cultures (Figure 7). Protoplast formation became sometime evident.

All this suggested that *L. muscarium* released diffusible inhibiting compounds (lytic enzymes and/or antibiotics) into the medium. This is typical of other mycoparasitic fungi such as *Trichoderma* spp. [48,50,51] or *Verticillium* spp. [40]. The low molecular weight chitinolytic enzyme, shown in Figure 2, could be a possible diffusible protein candidate; no tests had been carried out regarding possible production of antibiotics. It is worth nothing that the mycelial damages were also observed when crude preparation of *L. muscarium* CWDE was added to replace the fungus in the Petri dishes containing the host organisms.

However, after some time, aerial mycelium of *L. muscarium* went in contact with the hosts and, shortly thereafter, overwhelmed them. Samples from the contact zones of the plate co-cultures were observed under light microscopy and SEM. The contact between the Antarctic strain and the host was always very firm. Coiling was observed, but coils were less regularly developed around host hyphae if compared with other mycoparasites [49,50]. The typology of contact exerted by *L. muscarium* changed in relation to the host organism. With fungi, the it followed a typical sequence of events including attachment to the host, producing mechanical pressure, release of CWDE and penetration into the host mycelium [38,43].

**Figure 8:**
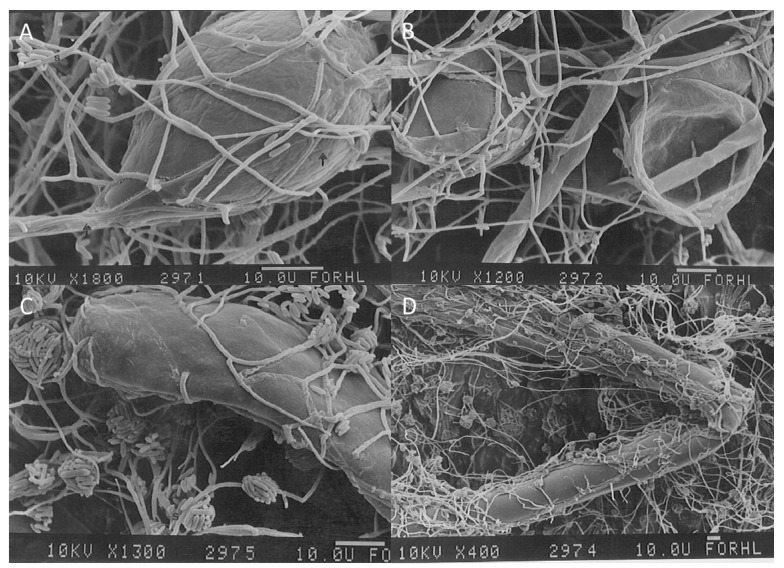
Top (**A**) Intermediate mycoparasitic stage of *L. muscarium* against *Phytophtora palmivora*: arrows show the contact between the thin mycelium of the Antarctic fungus and a *P. palmivora* sporangium; s = spores of *L. muscarium*. (**B**) Late mycoparasitic stage of *L. muscarium* against *P. palmivora*; the oomycete appeared completely overwhelmed by the fungus. Bottom. (**C**) Intermediate mycoparasitic stage of *L. muscarium* against *Mucor mucedo*: the Antarctic fungus (thin mycelium) establishes firm contact with the host (thick mycelium) and penetrates it. (**D**) Late mycoparasitic stage of *L. muscarium* against *M. mucedo*: the host appears completely overwhelmed. Extensive sporulation of *L. muscarium* is always recorded (modified from [19]).

With oomycetes, differently, no evident penetration but firm adhesion and possibly mechanical pressure occurred. Since the activity of glucanases by *L. muscarium* was lower than that of chitinolytic enzymes [19], lack of penetration could probably due to the production of CWDE having reduced action on the oomycetes cell wall with high glucan content. Moreover, *L. muscarium* mycelium sometime appeared almost fused with that of the host. In all cases, at the late stages of the interaction, complete destruction of the host, that appeared strongly deflated and invaded by the parasite, was observed. Abundant sporulation of *L. muscarium* was recorded also in this case (Figure 8). 

**Figure 9:**
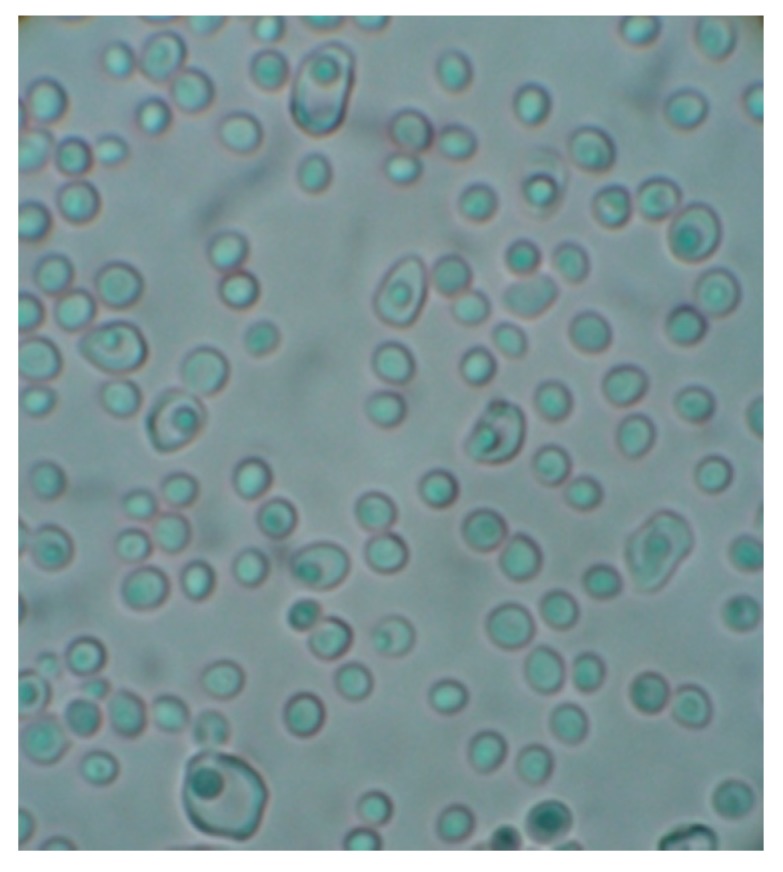
Light microscopy of *Candida vinaria* from a dual culture with *L. muscarium*. Most yeast cells appeared damaged and/or bigger than usual (Fenice *et al.*, unpublished results).

As for the action of the Antarctic fungus on yeasts, preliminary data (Fenice *et al.*, unpublished results) showed that, on co-cultures with *Candida vinaria*, the fungus behaviour was very similar to that described above: after some time, it overgrew the yeast. However, microscopic observations showed that the yeast was not completely destroyed by the fungus. Apparently, no contact was recorded between the two organisms, but many yeast cells appeared abnormally bigger and showed damaged cell walls. In addition, yeast cells were morphologically modified and traces of cell lysis were evident (Figure 9). Likely, the fungus released some CWDE affecting the yeasts but their concentration and/or composition was not appropriate to produce complete disruption. Further experiments are needed to understand possible parasitic role of *L. muscarium* against yeasts and its applied potentials. However, it is worth noting that the fungus has been proved to exert effective mycoparasitism against black yeasts (black meristematic fungi) such as *Cryomyces* spp. [52].

## 7. Conclusions

This work summarises more than 20 years of investigations carried out on the chitinolytic fungus *Lecanicillium muscarium* CCFEE 5003 isolated from samples collected in Continental Antarctica in the early nineties. In particular, we describe its ability to release a number of cold-active extracellular chitinolytic enzymes and its strong action as mycoparasite against fungi and oomycetes. This microorganism could be considered as one of the most promising “new” organisms isolated from extreme environments and deserves to be further investigated. Actually, due to its very interesting features, it would merit being exploited both at the industrial level, for the production of cold-tolerant enzymes, reuse of chitin-rich wastes, and for environmental applications, in biocontrol of pathogens due to its strong mycoparasitic activity. However, prior to introducing the Antarctic organism in different ecosystems, tests must be done to verify possible negative environmental and/or ecological effects.

## Figures and Tables

**Table 1:** Comparison of the partial chitinase sequence of *L. muscarium* CCFEE 5003 with other fungal chitinase as obtained by NCBI GenBank database query with BlastX. Only some of the best matches are shown.

Description	Query Coverage	Identity	Accession N°
Chitinase (*Metarhizium album*)	95%	42%	KHN99830.1
Chitinase (*Hirsutella thompsonii*)	94%	44%	AIT18903.1
Chitinase (*H. thompsonii*)	94%	44%	AIT18889.1
Endochitinase 33 (*Tolypocladium ophioglossoide*)	94%	43%	KND91481.1
Chitinase 33 (*Trichoderma virens*)	94%	41%	ACJ04784.1
Glycoside hydrolase family 18 (*T. reesei*)	94%	39%	XP_006961069.1
Glycoside hydrolase family 18 (*T. atroviride*)	94%	42%	XP_013947241.1
Endochitinase (*T. atroviride*)	94%	42%	ABO38127.1
Glycoside hydrolase family 18 (*T. virens*)	94%	41%	XP_013958614.1
Chitinase (*T. virens*)	94%	41%	ABP96986.1
Chitinase 3 (*Escovopsis weberi*)	94%	36%	KOS22932.1
Putative Chitinase (*Torrubiella hemipterigena*)	93%	50%	CEJ90418.1
Related to endochitinase (*Claviceps purpurea*)	93%	49%	CCE32650.1
